# Associations between LncRNA *MALAT1* Polymorphisms and Lymph Node Metastasis in Prostate Cancer

**DOI:** 10.3390/diagnostics11091692

**Published:** 2021-09-16

**Authors:** Ju-Chuan Hu, Shian-Shiang Wang, Ying-Erh Chou, Kun-Yuan Chiu, Jian-Ri Li, Chuan-Shu Chen, Sheng-Chun Hung, Cheng-Kuang Yang, Yen-Chuan Ou, Chen-Li Cheng, Chia-Yen Lin, Shun-Fa Yang

**Affiliations:** 1 Institute of Medicine, Chung Shan Medical University, Taichung 402, Taiwan; shelleycain525@gmail.com (J.-C.H.); sswdoc@yahoo.com.tw (S.-S.W.); intointo814@gmail.com (Y.-E.C.); fisherfishli@yahoo.com.tw (J.-R.L.); r2060d@yahoo.com.tw (C.-S.C.); weshong1118@gmail.com (S.-C.H.); ycou228@gmail.com (Y.-C.O.); cheng20011@gmail.com (C.-L.C.); 2 Division of Urology, Department of Surgery, Taichung Veterans General Hospital, Taichung 407, Taiwan; chiu37782002@yahoo.com (K.-Y.C.); yangck@vghtc.gov.tw (C.-K.Y.); 3 Division of Urology, Department of Surgery, Chiayi Branch, Taichung Veterans General Hospital, Chiayi 600, Taiwan; 4 Department of Applied Chemistry, National Chi Nan University, Nantou 545, Taiwan; 5 School of Medicine, Chung Shan Medical University, Taichung 402, Taiwan; 6 Department of Medical Research, Chung Shan Medical University Hospital, Taichung 402, Taiwan; 7 Department of Medicine and Nursing, Hung Kuang University, Taichung 433, Taiwan; 8 Department of Urology, Tung’s Taichung MetroHarbor Hospital, Taichung 433, Taiwan

**Keywords:** *MALAT1*, prostate cancer, polymorphism, metastasis

## Abstract

Current evidence elucidates that long noncoding RNA metastasis-associated lung adenocarcinoma transcript 1 (*MALAT1*) could regulate genetic expression and play a crucial role in both the diagnosis and prognosis of prostate cancer. Single-nucleotide polymorphisms (SNPs) of *MALAT1* could alter the oncogenesis in various cancers. However, the associations between *MALAT1* SNPs and prostate cancer have barely been investigated to date. This study included 579 patients with prostate cancer who received robotic-assisted radical prostatectomy at Taichung Veterans General Hospital from 2012 to 2017. Three SNPs of *MALAT1* were analyzed to identify the impacts of SNPs on the clinicopathologic features in Taiwanese prostate cancer. Our results show that patients with a polymorphic G allele at rs619586 had a significantly higher risk of being in an advanced Gleason grade group (AOR: 1.764; 95% CI: 1.011–3.077; *p* = 0.046). Moreover, individuals with at least one polymorphic A allele at *MALAT1* rs1194338 in the PSA >10 ng/mL group were positively associated with node-positive prostate cancer. In conclusion, *MALAT1* SNPs are significantly associated with the susceptibility to both advanced Gleason grade and nodal metastasis in prostate cancer. The presence of *MALAT1* SNPs rs619586 and rs1194338 seems to enhance oncogenesis in prostate cancer.

## 1. Introduction

In 2021, prostate cancer remains the most frequently diagnosed cancer and the second leading cause of mortality among American men [1]. Prostate-cancer-related death has diminished due to the extensive applications of prostate-specific antigen (PSA) testing; however, the overall downward trend of mortality stabilized after 2013, which is probably because of the recommendation of the United States Preventive Services Task Force (USPSTF) against PSA screening in 2012 and the rising diagnostic rates of late-stage prostate cancer [2]. There is a similar situation in Taiwan, although the overall incidence of prostate cancer is less frequent in Asia [3]. Less organ-confined prostate cancer and worse oncologic outcomes in the younger population have been reported in Taiwan [4,5]. Radical prostatectomy provides a definite treatment for prostate cancer, while concomitant pelvic lymph node dissection remains the gold standard for staging lymphadenopathy [6,7]. However, more aggressive operations come with the increased risk of surgical complications [8,9,10]. Therefore, more reliable prognostic predictors are still demanded in order to maintain the balance between overtreatment for prostate cancer and underestimation of cancer risk.

Tumorigenesis is an intricate process involving both genetic and epigenetic processes. For node-positive prostate cancer, both germline testing and somatic tumor testing for homologous recombination gene mutations (HRRm) and microsatellite instability (MSI) should be considered [11]. These molecular markers provide a precise guidance for further medical treatments, especially when prostate cancer progresses to castration-resistant status [12]. Long noncoding ribonucleic acids (lncRNAs) represent another potential diagnostic and therapeutic target in prostate cancer [13,14]. For example, prostate cancer antigen 3 (*PCA3*) gene testing used to enhance diagnostic accuracy is a well-known application of lncRNA in prostate cancer [13,15,16].

Metastasis-associated lung adenocarcinoma transcript 1 (*MALAT1*), located on 11q13 in humans, is an lncRNA originally identified as a prognostic marker in non-small-cell lung cancer (NSCLC) [17]. Compared to PSA screening in prostate cancer, the urinary *MALAT1* level is a more accurate diagnostic marker and helps to prevent 30.2–46.5% of unnecessary biopsies without missing any high-grade cancer in populations with PSA 4–10 ng/mL [18]. In addition to the diagnostic value, recent evidence indicates that *MALAT1* could also serve as a therapeutic target in prostate cancer [17]. *MALAT1* plays an essential role in the axis of the androgen receptor splicing variant 7 (AR-v7) and is found highly expressed in castration-resistant prostate cancer (CRPC) [19]. Furthermore, *MALAT1* serves as an RNA cofactor of the polycomb protein enhancer of zeste homolog 2 (EZH2) and subsequently enhances oncogenesis in CRPC [20]. The expression of *MALTA1* dramatically rises during the progression from hormone-sensitive to castration-resistant disease. Meanwhile, some studies have demonstrated that the silencing of *MALAT1* leads to a metabolic reprogramming in prostate cancer [19,20,21].

Single-nucleotide polymorphisms (SNPs), i.e., variants of a single nucleotide occurring at a specific genomic position, have been demonstrated as genetic markers of both incidence and prognosis in prostate cancer [22,23]. SNPs in lncRNA could modulate the expression of lncRNA and subsequently alter the susceptibility to prostate cancer [24]. Although more and more studies have evaluated the regulatory pathway of *MALAT1* in prostate cancer, scant data examining the association between *MALAT1* SNPs and prostate cancer are available to date. Our study aimed to access the potential roles of *MALAT1* polymorphisms in the clinicopathologic features of prostate cancer.

## 2. Materials and Methods

### 2.1. Study Population

From 2012 to 2017, a total of 579 consecutive patients with prostate adenocarcinoma was enrolled in this prospective study. The study was approved by the Institutional Review Board (IRB) of Taichung Veteran General Hospital (IRB No. CE19062A; 4 March 2019), and written informed consent was provided by each participant. These patients underwent robot-assisted radical prostatectomy with bilateral pelvic lymph node dissection. The medical profile included initial prostate-specific antigen (iPSA) when prostate cancer was diagnosed, clinical and pathologic tumor–node–metastasis (TNM) stage, pathologic Gleason grade group, D’Amico classification [25], and other pathologic findings.

The present study is an extension of our previous work exploring the predisposition of risk SNPs in prostate cancer and differentiating patients with extremely low oncologic risk from those traditionally defined as low risk in the D’Amico classification [25,26,27]. Thus, we divided the patients into three groups on the basis of their iPSA at diagnosis (iPSA ≤ 7 ng/mL, iPSA 7–10 ng/mL, and iPSA > 10 ng/mL) to analyze the role of *MALAT1* genotyping variation as a function of different levels of malignant potential in prostate cancer.

### 2.2. SNP Selection and DNA Extraction

Three *MALAT1* genetic variants, namely, rs3200401, rs619586, and rs1194338, were selected on the basis of data from the International HapMap Project dbSNP database and previous studies [28,29,30]. The allelic discrimination of *MALAT1* polymorphisms was evaluated using an ABI StepOne Real-Time PCR System (Applied Biosystems, Foster City, CA, USA) and analyzed by TaqMan Assay with SDS 3.0 software (Applied Biosystems, Foster City, CA, USA), as described in our previous study [31].

Venous blood was collected from all participants before radical prostatectomy. Whole-blood samples were then placed in ethylenediaminetetraacetic acid (EDTA)-coated vacutainers and centrifuged for further DNA extraction. Isolations of DNA from the buffy coats were performed using QIAamp DNA Blood Mini Kits (Qiagen, Valencia, CA, USA), according to the manufacturer’s protocol.

### 2.3. Statistical Analysis

Multiple logistic regression models were applied to access the adjusted odds ratio (AOR) with 95% confidence interval (CI) between *MALAT1* genotyping frequencies and three groups with different iPSA levels. The adjusted covariates included age at diagnosis, pathologic Gleason grade group, TNM stage, and some adverse pathologic features. A logistic regression model was used to estimate the odds ratio (OR) of each polymorphism in various clinical and pathologic presentations. Data were calculated using SAS statistical software (version 9.1; SAS Institute, Cary, NC, USA). The statistical significance was defined as a *p*-value <0.05.

## 3. Results

The distributions of clinical characteristics of enrolled subjects divided into three iPSA groups (153 cases with iPSA ≤ 7 ng/mL, 117 cases with iPSA 7–10 ng/mL, and 309 cases with iPSA > 10 ng/mL) are demonstrated in Table 1. There were higher incidences of Gleason grade 4 + 5, advanced clinical and pathologic TNM stage, adverse pathologic features, and high-risk D’Amico classification among patients with iPSA >10 ng/mL compared with the other two groups.

Table 2 demonstrates the distribution frequencies of the three *MALAT1* genotypes (namely, rs3200401, rs619586, and rs1194338) among 579 participants. The results showed that the percentage of patients carrying the homozygous polymorphic A allele in the rs1194338 polymorphism was significantly lower in both the iPSA 7–10 ng/mL group and the iPSA >10 ng/mL group compared to patients in the iPSA ≤7 ng/mL group. The frequency remained significantly lower in at least one polymorphic A allele (C/A + A/A genotype) at rs1194338 among participants with iPSA 7–10 ng/mL, although there was only a trend in participants with iPSA >10 ng/mL of having lower percentages of C/A + A/A genotype at the same loci. A nonsignificant higher frequency of A/G genotyping variant at rs619586 was also found in the iPSA >10 ng/mL group compared to patients in the iPSA ≤7 ng/mL group. In contrast, there was no obvious trend in polymorphism frequencies at rs3200401.

Subsequent analysis of SNP variants at rs619586 and rs1194338 to evaluate their potential relationship with the clinicopathologic features was undertaken in not only all participants (Table 3 and Table 4) but also focusing on patients with iPSA >10 ng/mL (Table 5). Patients with a polymorphic G allele at rs619586 had a significantly higher risk of advanced Gleason grade (AOR 1.764, 95% CI 1.011–3.077, *p* = 0.046) (Table 3). For patients with at least one polymorphic A allele at rs1194338, the risk of pathologic lymph node invasion was significantly increased regardless of iPSA level (AOR 3.348, 95% CI 1.501–7.469, *p* = 0.003) (Table 4). This significant association between rs1194338 and node-positive disease was mainly observed in cases with iPSA >10 ng/mL (AOR 3.452, 95% CI 1.350–8.826, *p* = 0.010) (Table 5). However, the presence of *MALAT1* genotyping variants was not significantly associated with biochemical recurrence (BCR) and overall survival (OS) in the present study.

## 4. Discussion

This is the first study to assess *MALAT1* polymorphisms and their clinicopathologic impact on Taiwanese men with operatable prostate cancer. Our results indicated that *MALAT1* SNP rs619586 is positively associated with advanced Gleason grade, while rs1194338 plays a part in lymph node metastasis, especially in patients with iPSA >10 ng/mL. Because *MALAT1* is considered an oncogene in genitourinary cancer [32], we believed that *MALAT1* genotyping variants might enhance its regulatory functions in prostate cancer, being subsequently responsible for cell proliferation and tumor invasion.

Lymph node invasion, one form of advanced prostate cancer, has a strong influence on adverse prognosis in either distant metastasis or cancer-specific survival [33,34]. Moreover, the number of positive lymph nodes can act as a powerful predictor for both biochemical recurrence and cancer-related death [35,36]. Thus, adjuvant hormonal therapy with/without radiotherapy is advocated for nodal metastasis prostate cancer in order to eliminate the occult micrometastasis, as well as improve cancer-specific and overall survival, especially in patients with high-risk prostate cancer [6,37,38,39,40].

However, there are no definite preoperative diagnosing tools to confirm nodal invasion. Traditional imaging tools including conventional computed tomography (CT) and magnetic resonance (MR) imaging are insensitive and inaccurate when the sizes of metastatic nodes are not prominent enough. Only some lymph node prediction nomograms are available today [41,42]. The current urologic guidelines suggest that various nomograms could be helpful in decision making for concurrent pelvic lymph node dissection during radical prostatectomy [11,43]. The parameters involved in these nomograms include age, iPSA, clinical stage, primary/secondary Gleason score, and the percentage of positive cores [42]. Unfortunately, there are still limitations to these nomograms, and further validation is persistently required [44,45,46,47].

Currently, pelvic lymph node dissection remains the gold standard for lymph node staging [6,7], while extended pelvic lymph node dissection is recommended for the correct staging in contrast to limited pelvic lymphadenectomy [48]. However, this invasive procedure could lead to various complications, although it provides both diagnostic and therapeutic benefits in intermediate-risk and high-risk prostate cancer [8,9,10]. Therefore, more meticulous but less invasive predictors are required to evaluate either the possibility of nodal metastasis or the indication for lymph node dissection.

Genetic polymorphisms could help the risk predictions of prostate cancer and might also interfere with oncologic prognosis in prostate cancer [22,49]. Our previous research showed that *carbonic anhydrate 9 (CA9)* polymorphism is associated with a 4.5-fold increased risk of lymph node metastasis while *growth arrest-specific 5 (GAS5)* SNPs play a protective role in nodal invasion (OR 0.545, *p* = 0.043) [50,51]. The present study demonstrates that *MALAT1* polymorphism rs1194338 leads to a 3.348-fold increased risk of node-positive prostate cancer. The susceptibility to node-positive prostate cancer was more remarkable in the PSA >10 ng/mL group (Table 5), although fewer patients in the PSA >10 ng/mL group carried at least one polymorphic A allele at rs1194338 compared to patients with a PSA level lower than 10 ng/mL (Table 2). With more information about how genotyping variants interfere with lymph node metastasis, we could develop an innovative lymph node nomogram in the future by taking account of the status of these SNPs to evaluate the indication of lymph node dissection and further requirement for adjuvant therapy.

The roles of *MALAT1* in tumorigenesis are complicated, as it can function as either a promoter or a suppressor in metastasis depending on the mechanism of action in different cancers [52]. Polymorphisms of *MALAT1* could alter its regulatory roles in the presplicing process and gene expression. Previous studies found that rs619586 A > G polymorphisms have a protective effect toward papillary thyroid cancer (OR = 0.76, 95% CI = 0.60–0.95, *p* = 0.017) and hepatocellular carcinoma (AOR = 0.29, 95% CI = 0.11–0.77, *p* = 0.013) [31,53]. Another *MALAT1* SNP rs1194338 was also identified as a protective factor toward colorectal cancer susceptibility (OR = 0.70, 95% CI = 0.49–0.99, *p* = 0.045) [54]. In stark contrast, these two SNPs were both associated with an aggressive tumorigenesis of prostate cancer in this cohort. Our results suggest that the regulatory pathway of *MALAT1* SNPs in prostate cancer might be different from other malignancies.

There were some limitations in the present study. Firstly, we only recruited patients who underwent radical prostatectomy. They were relatively younger with a mean age of 67.1 years old and with better functional performance, which allowed them to receive general anesthesia. These patients also had a less advanced and more operatable disease. These baseline characteristics might have served as protective confounders for prognosis and led to an insignificant result in biochemical recurrence (BCR), progression-free survival (PFS), and overall survival (OS). Secondly, the participants were assumed to have a hormone-sensitive status since they were treatment-naïve before prostatectomy. Therefore, this study only demonstrates the predictive impact of *MALAT1* SNPs in men with hormone-sensitive prostate cancer (HSPC). Compared to operatable HSPC, castration-resistant prostate cancer (CRPC) is a more heterogenous disease and presents with a different cell surface protein profile, which may alter the regulatory function of lncRNAs in oncogenesis. Further research assessing the potential effects of *MALAT1* SNPs on the castration process and therapeutic response will be conducted in the future. Thirdly, the follow-up period did not exceed 10 years, whereas life expectancy after the diagnosis of prostate cancer is 9.7 (95% CI 9.5–9.8) years in Taiwan [55]. Since prostate cancer is a relatively slow-progressing disease compared to other aggressive malignancies, this insufficient follow-up time might have led to the lack of difference in BCR, PFS, and OS. Furthermore, only Taiwanese men were accessed in this single-center study with a relatively small sample size. Hence, further research on larger cohorts and even the international multi-centers to confirm the association between these *MALAT1* SNPs and clinicopathological characteristics patients with prostate cancer needs to be conducted.

A previous meta-analysis concluded that the overexpression of *MALAT1* could predict lymph node metastasis in various types of cancer (pooled OR = 2.34, CI = 1.61–3.40, *p* < 0.001) [56]. However, only blood samples were collected in this cohort, and no cancer tissue was concomitantly harvested from the prostate specimen. Thus, we could only identify the relationship between *MALAT1* genotyping frequency and clinicopathologic characteristics. Further research is still required to confirm the definite associations between *MALAT1* expression and oncologic outcome.

## 5. Conclusions

*MALAT1* SNPs are positively associated with adverse pathologic features including nodal metastasis and advanced Gleason grades in operatable HSPC. With more information about risk SNPs in node-positive prostate cancer, a modified lymph node nomogram can be developed to enhance preoperative predictions and facilitate surgical plans in the era of precision medicine. Further studies examining the potential roles of lncRNA *MALAT1* in CRPC are also needed.

## Figures and Tables

**Table 1:** Distributions of demographical characteristics in 579 patients with prostate cancer.

Variable	PSA at Diagnosis (ng/mL)		
	≤7 (*n* = 153)	7–10 (*n* = 117)	>10 (*n* = 309)
Age at diagnosis (years)			
≤65	71 (46.4%)	61 (52.1%)	113 (36.6%)
>65	82 (53.6%)	56 (47.9%)	196 (63.4%)
Pathologic Gleason grade group			
1 + 2	123 (80.4%)	85 (72.6%)	152 (49.2%)
3 + 4 + 5	30 (19.6%)	32 (27.4%)	157 (50.8%)
Clinical T stage			
1 + 2	144 (94.1%)	109 (93.2%)	248 (80.3%)
3 + 4	9 (5.9%)	8 (6.8%)	61 (19.7%)
Pathologic T stage			
2	114 (74.5%)	72 (61.5%)	120 (38.8%)
3 + 4	39 (25.5%)	45 (38.5%)	189 (61.2%)
Pathologic N stage			
N0	146 (95.4%)	113 (96.6%)	384 (87.7%)
N1	7 (4.6%)	4 (3.4%)	38 (12.3%)
Extraprostatic extension			
No	107 (69.9%)	71 (60.7%)	148 (47.9%)
Yes	46 (30.1%)	46 (39.3%)	161 (52.1%)
Seminal vesicle invasion			
No	145 (94.8%)	99 (84.6%)	208 (67.3%)
Yes	8 (5.2%)	18 (15.4%)	101 (32.7%)
Perineural invasion			
No	53 (34.6%)	40 (34.2%)	62 (20.1%)
Yes	100 (65.4%)	77 (65.8%)	247 (79.9%)
Lymphovascular invasion			
No	145 (94.8%)	101 (86.3%)	236 (76.4%)
Yes	8 (5.2%)	16 (13.7%)	73 (23.6%)
D’Amico classification			
Low/intermediate risk	105 (68.6%)	85 (72.6%)	90 (29.1%)
High risk	48 (31.4%)	32 (27.4%)	219 (70.9%)
Total score upgrade			
No	87 (56.9%)	74 (63.2%)	204 (66.0%)
Yes	66 (43.1%)	43 (36.8%)	105 (34.0%)
Grade group upgrade			
No	84 (54.9%)	72 (61.5%)	187 (60.5%)
Yes	69 (45.1%)	45 (38.5%)	122 (39.5%)

**Table 2:** Distribution frequency of *MALAT1* genotypes in 579 patients with prostate cancer.

Variable	PSA at Diagnosis (ng/mL)				
	≤7 (*n* = 153)	7–10 (*n* = 117)	>10 (*n* = 309)	AOR (95% CI) ^a^	AOR (95% CI) ^b^
rs3200401					
CC	89 (58.2%)	76 (65.0%)	210 (68.0%)	1.00	1.00
CT	57 (37.3%)	35 (29.9%)	89 (28.8%)	0.760 (0.440–1.315) *p* = 0.327	0.754 (0.464–1.224) *p* = 0.253
TT	7 (4.5%)	6 (5.1%)	10 (3.2%)	0.974 (0.299–3.168) *p* = 0.965	0.641 (0.205–2.006) *p* = 0.445
CT + TT	64 (41.8%)	41 (35.0%)	99 (32.0%)	0.785 (0.465–1.326) *p* = 0.366	0.741 (0.464–1.183) *p* = 0.209
rs619586					
AA	138 (90.2%)	105 (89.7%)	250 (80.9%)	1.00	1.00
AG	15 (9.8%)	12 (10.3%)	59 (19.1%)	1.154 (0.506–2.630) *p* = 0.733	1.942 (0.972–3.881) *p* = 0.060
GG	0 (0%)	0 (0%)	0 (0%)	---	---
AG + GG	15 (9.8%)	12 (10.3%)	59 (19.1%)	1.154 (0.506–2.630) *p* = 0.733	1.942 (0.972–3.881) *p* = 0.060
rs1194338					
CC	50 (32.7%)	55 (47.0%)	139 (45.0%)	1.00	1.00
CA	70 (45.8%)	52 (44.4%)	128 (41.4%)	0.703 (0.406–1.218) *p* = 0.209	0.758 (0.457–1.256) *p* = 0.283
AA	33 (21.6%)	10 (8.5%)	42 (13.6%)	0.231 (0.098–0.544) *p* = 0.001	0.459 (0.237–0.890) *p* = 0.021
CA + AA	103 (67.3%)	62 (53.0%)	170 (55.0%)	0.545 (0.324–0.916) *p* = 0.022	0.661 (0.412–1.060) *p* = 0.086

The adjusted odds ratios (AORs) with their 95% confidence intervals (CIs) were estimated by multiple logistic regression models after controlling for age at diagnosis, pathologic Gleason grade group, clinical T stage, pathologic T stage, pathologic N stage, extraprostatic extension, seminal vesicle invasion, perineural invasion, lymphovascular invasion, and D’Amico classification. ^a^ AORs with their 95% CIs were calculated between patients with PSA level ≤7 ng/mL and PSA level 7–10 ng/mL; ^b^ AORs with their 95% CIs were calculated between patients with PSA level ≤7 ng/mL, and PSA level >10 ng/mL.

**Table 3:** Odds ratio (OR) and 95% confidence interval (CI) of clinical status and *MALAT1* rs619586 genotypic frequencies in 579 patients with prostate cancer.

Variable	Genotypic Frequencies			
rs619586	AA (*N* = 493)	AG + GG (*N* = 86)	AOR (95% CI)	*p*-Value
Pathologic Gleason grade group				
1 + 2	317 (64.3%)	43 (50.0%)	1.00	*p* = 0.046 *
3 + 4 + 5	176 (35.7%)	43 (50.0%)	1.764 (1.011–3.077)	
Clinical T stage				
1 + 2	426 (86.4%)	75 (87.2%)	1.00	*p* = 0.605
3 + 4	67 (13.6%)	11 (12.8%)	0.814 (0.374–1.773)	
Pathologic T stage				
2	265 (53.8%)	41 (47.7%)	1.00	*p* = 0.748
3 + 4	228 (46.2%)	45 (52.3%)	0.870 (0.373–2.031)	
Pathologic N stage				
N0	447 (90.7%)	83 (96.5%)	1.00	*p* = 0.086
N1	46 (9.3%)	3 (3.5%)	0.389 (0.125–1.324)	
Extraprostatic extension				
No	280 (56.8%)	46 (53.5%)	1.00	*p* = 0.838
Yes	213 (43.2%)	40 (46.5%)	0.923 (0.427–1.996)	
Seminal vesicle invasion				
No	389 (78.9%)	63 (73.3%)	1.00	*p* = 0.504
Yes	104 (21.1%)	23 (26.7%)	1.276 (0.625–2.606)	
Perineural invasion				
No	138 (28.0%)	17 (19.8%)	1.00	*p* = 0.339
Yes	355 (72.0%)	69 (80.2%)	1.363 (0.722–2.573)	
Lymphovascular invasion				
No	412 (83.6%)	70 (81.4%)	1.00	*p* = 0.604
Yes	81 (16.4%)	16 (18.6%)	1.210 (0.590–2.481)	
D’Amico classification				
Low/intermediate risk	246 (49.9%)	34 (39.5%)	1.00	*p* = 0.104
High risk	247 (50.1%)	52 (60.5%)	1.486 (0.878–2.516)	
Total score upgrade				
No	313 (63.5%)	52 (60.5%)	1.00	*p* = 0.931
Yes	180 (36.5%)	34 (39.5%)	1.041 (0.413–2.628)	
Grade group upgrade				
No	296 (60.0%)	47 (54.7%)	1.00	*p* = 0.913
Yes	197 (40.0%)	39 (45.3%)	1.053 (0.417–2.656)	

The adjusted odds ratios (AORs) with their 95% confidence intervals (CIs) were estimated by multiple logistic regression models after controlling for pathologic Gleason grade group, clinical T stage, pathologic T stage, pathologic N stage, extraprostatic extension, seminal vesicle invasion, perineural invasion, lymphovascular invasion, D’Amico classification, total score upgrade, and Grade group upgrade. * *p* < 0.05 was considered statistically significant.

**Table 4:** Odds ratio (OR) and 95% confidence interval (CI) of clinical status and *MALAT1* rs1194338 genotypic frequencies in 579 patients with prostate cancer.

Variable	Genotypic Frequencies			
rs1194338	CC (*N* = 244)	CA + AA (*N* = 335)	AOR (95% CI)	*p*-Value
Pathologic Gleason grade group				
1 + 2	151 (61.9%)	209 (62.4%)	1.00	*p* = 0.918
3 + 4 + 5	93 (38.1%)	126 (37.6%)	0.978 (0.644–1.487)	
Clinical T stage				
1 + 2	212 (86.9%)	289 (86.3%)	1.00	*p* = 0.981
3 + 4	32 (13.1%)	46 (13.7%)	0.993 (0.561–1.757)	
Pathologic T stage				
2	123 (50.4%)	183 (54.6%)	1.00	*p* = 0.340
3 + 4	121 (49.6%)	152 (45.4%)	0.738 (0.395–1.377)	
Pathologic N stage				
N0	232 (95.1%)	298 (89.0%)	1.00	*p* = 0.003 *
N1	12 (4.9%)	37 (11.0%)	3.348 (1.501–7.469)	
Extraprostatic extension				
No	138 (56.6%)	188 (56.1%)	1.00	*p* = 0.189
Yes	106 (43.4%)	147 (43.9%)	1.479 (0.825–2.651)	
Seminal vesicle invasion				
No	188 (77.0%)	264 (78.8%)	1.00	*p* = 0.749
Yes	56 (23.0%)	71 (21.2%)	0.915 (0.532–1.575)	
Perineural invasion				
No	53 (21.7%)	102 (30.4%)	1.00	*p* = 0.028 *
Yes	191 (78.3%)	233 (69.6%)	0.614 (0.397–0.949)	
Lymphovascular invasion				
No	203 (83.2%)	279 (83.3%)	1.00	*p* = 0.408
Yes	41 (16.8%)	56 (16.7%)	0.785 (0.442–1.393)	
D’Amico classification				
Low/intermediate risk	113 (46.3%)	167 (49.9%)	1.00	*p* = 0.399
High risk	131 (53.7%)	168 (50.1%)	0.848 (0.577–1.244)	
Total score upgrade				
No	145 (59.4%)	220 (65.7%)	1.00	*p* = 0.073
Yes	99 (40.6%)	115 (34.3%)	0.508 (0.243–1.065)	
Grade group upgrade				
No	140 (57.4%)	203 (60.6%)	1.00	*p* = 0.188
Yes	104 (42.6%)	132 (39.4%)	1.642 (0.784–3.437)	

The adjusted odds ratios (AORs) with their 95% confidence intervals (CIs) were estimated by multiple logistic regression models after controlling for pathologic Gleason grade group, clinical T stage, pathologic T stage, pathologic N stage, extraprostatic extension, seminal vesicle invasion, perineural invasion, lymphovascular invasion, D’Amico classification, total score upgrade, and Grade group upgrade. * *p* < 0.05 was considered statistically significant.

**Table 5:** Odds ratio (OR) and 95% confidence interval (CI) of clinical status and *MALAT1* rs1194338 genotypic frequencies in 309 patients with prostate cancer with a PSA concentration >10 ng/mL.

Variable	Genotypic Frequencies			
rs1194338	CC (*N* = 139)	CA + AA (*N* = 170)	AOR (95% CI)	*p*-Value
Pathologic Gleason grade group				
1 + 2	69 (49.6%)	83 (48.8%)	1.00	*p* = 0.943
3 + 4 + 5	70 (50.4%)	87 (51.2%)	0.980 (0.567–1.695)	
Clinical T stage				
1 + 2	111 (79.9%)	137 (80.6%)	1.00	*p* = 0.594
3 + 4	28 (20.1%)	33 (19.4%)	0.835 (0.430–1.622)	
Pathologic T stage				
2	54 (38.8%)	66 (38.8%)	1.00	*p* = 0.920
3 + 4	85 (61.2%)	104 (61.2%)	0.955 (0.388–2.351)	
Pathologic N stage				
N0	129 (92.8%)	142 (83.5%)	1.00	*p* = 0.010 *
N1	10 (7.2%)	28 (16.5%)	3.452 (1.350–8.826)	
Extraprostatic extension				
No	69 (49.6%)	79 (46.5%)	1.00	*p* = 0.393
Yes	70 (50.4%)	91 (53.5%)	1.437 (0.625–3.302)	
Seminal vesicle invasion				
No	94 (67.6%)	114 (67.1%)	1.00	*p* = 0.992
Yes	45 (32.4%)	56 (32.9%)	1.003 (0.531–1.897)	
Perineural invasion				
No	22 (15.8%)	40 (23.5%)	1.00	*p* = 0.057
Yes	117 (84.2%)	130 (76.5%)	0.501 (0.254–1.012)	
Lymphovascular invasion				
No	107 (77.0%)	129 (75.9%)	1.00	*p* = 0.354
Yes	32 (23.0%)	41 (24.1%)	0.722 (0.363–1.438)	
D’Amico classification				
Low/intermediate risk	38 (27.3%)	52 (30.6%)	1.00	*p* = 0.473
High risk	101 (72.7%)	118 (69.4%)	0.815 (0.466–1.425)	
Total score upgrade				
No	87 (62.6%)	117 (68.8%)	1.00	*p* = 0.203
Yes	52 (37.4%)	53 (31.2%)	0.552 (0.221–1.378)	
Grade group upgrade				
No	82 (59.0%)	105 (61.8%)	1.00	*p* = 0.322
Yes	57 (41.0%)	65 (38.2%)	1.582 (0.639–3.916)	

The adjusted odds ratios (AORs) with their 95% confidence intervals (CIs) were estimated by multiple logistic regression models after controlling for pathologic Gleason grade group, clinical T stage, pathologic T stage, pathologic N stage, extraprostatic extension, seminal vesicle invasion, perineural invasion, lymphovascular invasion, D’Amico classification, total score upgrade, and Grade group upgrade. * *p* < 0.05 was considered statistically significant.

## Data Availability

The datasets generated for this study are available on request to the corresponding authors.
